# Supplementary material for: COVID-19 Intensive Care—Evaluation of Public Information Sources and Current Standards of Care in German Intensive Care Units: A Cross Sectional Online Survey on Intensive Care Staff in Germany
Source: Healthcare (Basel). 2022 Jul 15;10(7):1315. doi: 10.3390/healthcare10071315 (PMC9319015; doi:10.3390/healthcare10071315)
Supplement: Supplementary file 1 [file healthcare-10-01315-s001.zip › Table S1.pdf]

Table S1 CHERRIES

Item category	Checklist Item	Reported
Design	Describe survey design	✓
IRB (Institutional Review Board) approval and informed consent process	IRB approval	✓
	Informed consent	✓
Development and pre-testing	Development and testing	✓
Recruitment process and description of the sample having access to the questionnaire	Open survey vs closed survey	✓
	Contact mode	✓
	Advertising the survey	✓
Survey administration	Web/E-mail	✓
	Context	✓
	Mandatory/voluntary	✓
	Incentives	✓
	Time/Date	✓
	Randomization of items or questionnaires	✓
	Adaptive questioning	✓
	Number of Items	✓
	Number of screens (pages)	✓
Response rates	Unique site visitor	n/a
	View rate (Ratio of unique survey visitors/unique site visitors)	n/a
	Participation rate (Ratio of unique visitors who agreed to participate/unique first survey page visitors)	n/a
	Completion rate (Ratio of users who finished the survey/users who agreed to participate)	✓
Preventing multiple entries from the same individual	Cookies used	✓
	IP Check	✓
	Log file analysis	✓
	Registration	✓
Analysis	Handling of incomplete questionnaires	✓
	Questionnaires submitted with an atypical timestamp	n/a
	Statistical correction	✓
