# Supplementary material for: COVID-19 Intensive Care—Evaluation of Public Information Sources and Current Standards of Care in German Intensive Care Units: A Cross Sectional Online Survey on Intensive Care Staff in Germany
Source: Healthcare (Basel). 2022 Jul 15;10(7):1315. doi: 10.3390/healthcare10071315 (PMC9319015; doi:10.3390/healthcare10071315)
Supplement: Supplementary file 1 [file healthcare-10-01315-s001.zip › Table S2.pdf]

Table S2 questionnaire

Questionnaire, notes for reply are highlighted in italics,
* for questions with multiple answers possible,
([a]) question identical to 2020 survey

What type of facility do you work in?		
o Primary care/standard care hospital (service Level 1)<br>o Hospital with special care (service level 2)	o specialist clinic<br>o maximum care hospital (service level 3)<br>o other (free text)	
**Have you ever treated COVID Patients in the ICU?**		
o yes	o no	
**To which professional group do you belong?**		
o medical resident<br>o specialist	o intensive care nurse<br>o nurse<br>o other health professionals	
**Do you perform a ward management function with setting of treatment/care plan?**		
o yes	o no	
**Do you currently have an established standard of care for patients with COVID-19 pneumonia?**		
o yes	o no	o I don’t know
**Were you a collaborator in the CEOsys project?**		
o yes	o no	
**To which of the following age categories do you belong?**		
o <20<br>o 21-25<br>o 26-30<br>o 31-35	o 36-40<br>o 41-45<br>o 46-50<br>o >50	
**Which of the following sources of information about Covid-19 therapy do you use?***		
o Everyday-media (e.g. radio, television) → please name which specific sources you use<br>o Social Media → please name which specific sources you use<br>o Medical journals → please name which specific sources you use<br>o Websites of professional societies → please name which specific sources you use<br>o Newsletter/Emails → please name which specific sources you use utzen<br>o Medical information portals → please name which specific sources you use<br>o Free Open Access medial Eduacation – FOAM (z.B. emcrit.org, nerdfallmedizin.blog) → please name which specific sources you use<br>o In-house Standard Operation Procedures or procedural instructions		

Please rate the quality of the following sources of information on COVID-19 therapy?*<br>0=lowest quality to 10=very high quality, please also indicate if you do not using the rated option<br>*NRS from 0 to 10 plus "I do not know or do not know enough to evaluate the quality."*		
		I don't know or not good enough to evaluate the quality.
o Everyday-media (e.g. radio, TV)	0 1 2 3 4 5 6 7 8 9 10	o
o Social Media	0 1 2 3 4 5 6 7 8 9 10	o
o Medical journals	0 1 2 3 4 5 6 7 8 9 10	o
o Websites of professional societies	0 1 2 3 4 5 6 7 8 9 10	o
o Newsletter/Emails	0 1 2 3 4 5 6 7 8 9 10	o
o Medical information portals	0 1 2 3 4 5 6 7 8 9 10	o
o Free Open Access medial Eduacation – FOAM (z.B. emcrit.org, nerdfallmedizin.blog)	0 1 2 3 4 5 6 7 8 9 10	o
o In-house Standard Operation Procedures or procedural instructions	0 1 2 3 4 5 6 7 8 9 10	o

Which organizations (source of information) do you consider as most trustworthy for information regarding medical therapy for COVID-19? ([a])max.3***

- o Cochrane Collaboration
- o German network evidence-based medicine (EbM-Netzwerk)
- o Professional societies (z.B. DIVI, DGAI, DGIIN)
- o National Health Care Guidelines Programme (Cooperation of Bundesärztekammer, KBV, AWMF)
- o World Health Organisation (WHO)
- o Robert-Koch-Institut (RKI)
- o Federal Center for Health Education (Bundeszentrale für gesundheitliche Aufklärung - BZgA)
- o others → free text

Do you use the S3- Guideline - Recommendations for inpatient therapy of patients with COVID-19? *(filter question)*

o yes   o no

If yes: How do you assess the applicability of the guideline?

o very bad (explanatory note)	o rather bad (explanatory note)	o undecided	o rather good	o very good

If no: What barriers make it difficult for you to use the guideline? **max.3*

- o I don't have enough time to deal with the guidelines in my daily work.
- o the use is not encouraged by my superiors.
- o I have no experience in working with guidelines.
- o access to the guidelines is too complicated.
- o the contents of the guidelines are in my opinion not practicable in everyday life (e.g. too undifferentiated, not applicable to the individual).
- o the contents of the guidelines are not well adapted to my target group (e.g. with regard to prior knowledge, relevance).
- o Guidelines are too long.
- o I don't know where or how to access current guidelines.
- o others -> free text

Do you use the CEOsys website www.covid-evidenz.de ? *(filter question)*

- o yes
- o no

If yes: Would you recommend the website www.covid-evidenz.de?

- o Yes (free text: reasons)
- o no (free text: reasons)
- o I don't know

If no: What barriers make it difficult for you to use the www.covid-evidenz.de website? **max.3*

- o I don't have enough time to deal with the website in my daily work.
- o the use is not encouraged by my superiors.
- o i have no experience in using the website.
- o access to the website is too complicated
- o the contents of the website are, in my opinion, not practicable in everyday life (e.g. too undifferentiated, not applicable to the individual).
- o the contents of the guideline are not well adapted to my target group (e.g. with regard to prior knowledge, relevance).
- o the website isn't user-friendly
- o i don't know the website
- o other (free text)

Do you use the MagicApp? (A digital platform for creating and publishing evidence ecosystems from the Magic Evidence Foundation). *(filter question)*

- o yes
- o no

If yes: Would you recommend the use of the MagicApp?

- o Yes
- o no (free text: reasons)
- o I don't know

If yes: How do you assess the applicability of the information provided there?

- o very bad (free text: reasons)
- o rather bad (free text: reasons)
- o undecided
- o rather good
- o very good

If no: What barriers make it difficult for you to use the MagicApp? **max.3*

- o I don't have enough time to deal with the website in my daily work.
- o the use is not encouraged by my superiors.
- o i have no experience in using the app.
- o access is too complicated (technical requirements).
- o the contents are in my opinion not practicable in everyday life (e.g. too undifferentiated, not applicable to the individual).
- o the contents of the guidelines are not well adapted to my target group (e.g. with regard to prior knowledge, relevance).
- o MagicApp isn't user-friendly
- o I don't know where or how to access the app.
- o other (free text)

Would you need to make a decision on how to proceed? What criteria would you use for discontinuing NIV ventilation in COVID-19 patients? ([a]) *

- Consciousness disorder
- Respiratory rate
- Clinical assessment of the respiratory work
- Rapid-shallow-breathing-Index
- CO2 elimination disorder
- Horovitz/oxygenation index
- Work-of-breathing
- I don't know

What is the standard dosage of thromboembolism prophylaxis medication for patients you have to treat in the ICU for COVID-19 pneumonia?

- prophylactic
- semi-therapeutic (filter question)
- therapeutic (filter question)

If Semi-therapeutic/ Therapeutic anticoagulation: Do you control this dosage based on laboratory coagulation controls?

- Yes
- no
- I don't know

What COVID-19 specific medications are you currently using for therapy? *

- Corticosteroid (e.g. Dexamethason)
- Remdesivir
- JAK Inhibitors (e.g. Baricitinib)
- Specific antibodies
- Reconvalescent plasma
- Ivermectin
- Vitamin D
- Hydroxychloroquin
- IL-6 receptor blockers (e.g. Tocilizumab, Sarilumab)
- Lopinavir/Ritonavir
- other (free text)

Follow-up for selected medications: What is the rational for the decision to use this therapy?

- S3-guidelines inpatient treatment COVID-19
- own literature research
- determined by superiors
- good experience with this medication
- I don't know

Item from 2020 Survey

Severely ill COVID-19 patients should be treated with therapeutic anticoagulation/blood dilution.

- I do not agree at all
- I rather disagree
- I rather agree
- I fully agree
- I don't know
