# Supplementary material for: COVID-19 Intensive Care—Evaluation of Public Information Sources and Current Standards of Care in German Intensive Care Units: A Cross Sectional Online Survey on Intensive Care Staff in Germany
Source: Healthcare (Basel). 2022 Jul 15;10(7):1315. doi: 10.3390/healthcare10071315 (PMC9319015; doi:10.3390/healthcare10071315)
Supplement: Supplementary file 1 [file healthcare-10-01315-s001.zip › Table S3.pdf]

Table S3

Age characteristics for participants

| age group | medical residents (n=26) | | specialists (n=50) | | nurses (n=20) | | intensive care nurses (n=31) | | total (n=127) | |
|---|---|---|---|---|---|---|---|---|---|---|
| | **n** | **%** | **n** | **%** | **n** | **%** | **n** | **%** | **n** | **%** |
| <20 - 30 | 12 | 46.2 | - | - | 10 | 50.0 | 2 | 6.5 | 24 | 19.9 |
| 31 - 40 | 14 | 53.8 | 17 | 34.0 | 5 | 25.0 | 6 | 19.4 | 42 | 33.1 |
| 41 - 50 | - | - | 19 | 38.0 | 3 | 15.0 | 14 | 45.2 | 36 | 28.3 |
| >50 | - | - | 14 | 28.0 | 2 | 10.0 | 9 | 29.0 | 25 | 19.7 |

Group differences in quality assessment between users and non-users (Mann-Whitney-U-Test)

| | *U* | *Z* | *p* | *r* |
|---|---|---|---|---|
| social media (n=98) | 147.00 | -4.81 | <.001* | -0.49 |
| everyday-media (e.g. Radio, TV) (n=116) | 811.000 | -4.60 | <.001* | -0.43 |
| newsletter/ email (n=94) | 470.000 | -3.66 | <.001* | -0.38 |
| FOAM (n=56) | 166.500 | -3.366 | <.001* | -0.45 |
| medical information portals (n=87) | 731.000 | -1.78 | .075 | -0.19 |
| websites of the scientific medical societies (n=100) | 830.500 | -2.51 | .012 | -0.25 |
| medical journals (n=106) | 812.000 | -2.76 | .006* | -0.27 |
| inhouse SOP's/procedural instructions (n=119) | 886.000 | -1.27 | .206 | -0.12 |

* significant p<.05

Percentage of coagulation dosing according to professions

| coagulation | physicians (n=64) | | nursing staff (n=38) | | total (n=102) | |
|---|---|---|---|---|---|---|
| | n | % | n | % | n | % |
| prophylactic dosing | 36 | 56.2 | 16 | 42.1 | 52 | 51.0 |
| semitherapeutic dosing | 16 | 25.0 | 8 | 21.1 | 24 | 23.5 |
| therapeutic dosing | 12 | 18.8 | 14 | 36.8 | 26 | 25.5 |
